# Thoughtseeds: A Hierarchical and Agentic Framework for Investigating Thought Dynamics in Meditative States

**DOI:** 10.3390/e27050459

**Published:** 2025-04-24

**Authors:** Prakash Chandra Kavi, Gorka Zamora-López, Daniel Ari Friedman, Gustavo Patow

**Affiliations:** 1 Center for Brain and Cognition, Universitat Pompeu Fabra, 08005 Barcelona, Spain; gorka.zamora@upf.edu (G.Z.-L.); gustavo.patow@udg.edu (G.P.); 2 Active Inference Institute, Davis, CA 95616, USA; daniel@activeinference.institute; 3 Department of Computer Science, Applied Mathematics and Statistics, University of Girona, 17003 Girona, Spain

**Keywords:** content of consciousness, embodied cognition, Markov blanket, meditation, Vipassana, meta-cognition, thoughtseed, active inference, global workspace

## Abstract

The Thoughtseeds Framework introduces a novel computational approach to modeling thought dynamics in meditative states, conceptualizing thoughtseeds as dynamic attentional agents that integrate information. This hierarchical model, structured as nested Markov blankets, comprises three interconnected levels: (i) knowledge domains as information repositories, (ii) the Thoughtseed Network where thoughtseeds compete, and (iii) meta-cognition regulating awareness. It simulates focused-attention Vipassana meditation via rule-based training informed by empirical neuroscience research on attentional stability and neural dynamics. Four states—breath_control, mind_wandering, meta_awareness, and redirect_breath—emerge organically from thoughtseed interactions, demonstrating self-organizing dynamics. Results indicate that experts sustain control dominance to reinforce focused attention, while novices exhibit frequent, prolonged mind_wandering episodes, reflecting beginner instability. Integrating Global Workspace Theory and the Intrinsic Ignition Framework, the model elucidates how thoughtseeds shape a unitary meditative experience through meta-awareness, balancing epistemic and pragmatic affordances via active inference. Synthesizing computational modeling with phenomenological insights, it provides an embodied perspective on cognitive state emergence and transitions, offering testable predictions about meditation skill development. The framework yields insights into attention regulation, meta-cognitive awareness, and meditation state emergence, establishing a versatile foundation for future research into diverse meditation practices (e.g., Open Monitoring, Non-Dual Awareness), cognitive development across the lifespan, and clinical applications in mindfulness-based interventions for attention disorders, advancing our understanding of the nature of mind and thought.

## 1. Introduction

*“The most intelligent minds are those that can entertain an idea without necessarily believing in it”.—Aristotle*

### 1.1. Embodied Cognition: An Evolutionary and Variational Free Energy Perspective

Embodied cognition [1,2] emphasizes the dynamic interaction between the brain, body, and environment, highlighting the critical roles of sensorimotor processes, bodily states, and environmental feedback in shaping cognitive processes. This framework is enriched by insights from evolutionary biology [3], which illustrates how living systems actively shape their surroundings through niche construction, tool use, and social interactions. The interplay is further shaped by evolutionary mechanisms [4,5], alongside epigenetic factors [6] and cultural inheritance [7]. Additionally, living systems actively regulate their environmental interactions through actions and perceptions [8,9].

Following this perspective, autopoiesis describes the self-organizing nature of living systems [10], enabling them to maintain a non-equilibrium steady state (NESS) through continuous exchange of energy and matter with their surroundings [11]. To maintain this state, organisms must actively minimize surprise and engage in adaptive behavior [12,13]. The Free Energy Principle (FEP) provides a framework for elucidating how living systems adapt by minimizing surprise or variational free energy [14,15]. Through active inference, living systems actively sample and shape their environments to align their predictions with sensory data, thereby ensuring their continued existence and minimizing entropy [16,17]. This process involves perceiving and acting upon affordances of the environment, or opportunities for action [17]. The Markov blanket concept facilitates computational autonomy while mediating environmental interactions [15,16].

FEP’s scale-free modeling approach integrates evolutionary and cognitive dynamics [18,19]. Furthermore, the Hierarchically Mechanistic Mind (HMM) hypothesis conceptualizes the brain as an adaptive control system that minimizes free energy through recursive interactions between neurocognitive processes, which emerge as a result of evolutionary pressures and self-organization [20].

### 1.2. Neuronal Packets (NPs) Under the Free Energy Principle

The brain’s sparse architecture supports localized functional units for specific cognitive tasks [21,22], reflecting “ascending scales of canonical microcircuits” [23,24,25]. It is guided by evolutionary priors that favor adaptive neural architectures [26], as evidenced by the distinct microcircuits of the visual cortex for color, motion, and form [27,28].

Neuronal representations extend beyond single neurons or simple neuronal assemblies, encompassing concepts such as Hebbian cell assemblies (cognits) [29], grid-like representations of conceptual knowledge [30], and memory engrams [31]. Building on these foundational ideas of structured and dynamic neuronal representations, the Neuronal Packet Hypothesis (NPH) [32,33,34] posits that neuronal packets (NPs) serve as fundamental units of neuronal representation in the brain [32].

Under the Active Inference framework, the brain reduces surprise by minimizing variational free energy (VFE), a measure of prediction error [15]. Neuronal packets (NPs) are ensembles of neurons that self-organize by competing to lower VFE, specializing in features like color or sound [33,35]. When activated, NPs emit signals, forming transient Markov blankets—temporary boundaries grouping NPs with aligned predictions [22,33]. From this, superordinate ensembles (SEs) emerge as initially transient entities, integrating NP signals to represent complex, context-dependent concepts like “a red ball” [33]. Through repeated learning and synaptic plasticity, SEs can stabilize, their Markov blankets strengthening to form enduring structures nested across scales. The shared generative model, a collective predictive framework co-created by NPs and SEs, acts as a dynamic map of expected patterns [34], refined by bottom-up signals (e.g., NPs reporting “red”) and top-down predictions (e.g., SEs expecting “a ball”). NPs’ Markov blankets nest within SEs’, enabling a modular, hierarchical system for multi-scale knowledge representation. Active Inference frames these as tools for guiding actions—like catching a ball—to minimize surprise [15,32].

Key properties of NPs and SEs are summarized in Table 1.

The paper is structured as follows: Section 2 introduces the Thoughtseeds Framework, building upon the concepts we discussed in this section. Section 3 applies this framework to model Vipassana meditation, detailing the learning process used to parameterize the model. Section 4 presents the results of simulating meditation sessions with the trained model, followed by a discussion in Section 5.

## 2. Introduction: Thoughtseeds Framework

### 2.1. Thoughtseeds Hypothesis

The brain’s cognitive repertoire is vast, posing a significant challenge in understanding how thoughts form and shape our experiences. To address this complexity, we introduce the Thoughtseed Framework, a research tool designed to investigate meditative states and mind-wandering episodes as a starting point to explore thought dynamics. These states are well-suited for study, as their phenomenologies have been thoroughly examined from both empirical perspectives (through neuroscientific research) [36,37,38,39,40] and subjective viewpoints (via reports from advanced meditators and traditional meditation literature) [41,42,43]. This dual approach aligns closely with embodied cognition principles, which posit that cognition emerges from the dynamic interplay of mind, body, and environment [1,41]. By focusing on these well-documented mental states, the Thoughtseed Framework offers a systematic approach to probing the origins and transformations of thoughts.

The framework builds upon robust theoretical foundations, including:

- **Neuronal Packets** (NPs) [32,33,34], which represent *fundamental units of neuronal representation* within the **Free Energy Principle** (FEP) [15,16,17,18], a computational model of how the brain minimizes uncertainty;
- **Global Workspace Theory** (GWT) [44,45,46,47], which describes how information becomes consciously accessible in the brain;
- **The Intrinsic Ignition Framework** [48], which explores spontaneous neural events driving cognition.

To demonstrate its practical utility, we present a proof-of-concept simulation of the Vipassana meditation process, comparing novice and expert meditators. Vipassana, a practice emphasizing mindful awareness of bodily sensations and mental events, serves as an ideal test case. This simulation not only demonstrates the framework’s applicability but also aligns with empirical findings on meditative states [36,38], elucidating the interplay of attention, thought formation, and subjective experience. By integrating computational modeling with phenomenological insights, the Thoughtseeds Framework provides a robust tool for investigating thought dynamics, offering a biologically plausible perspective to enhance our understanding of meditative states—particularly in the contexts of meditation and mind-wandering—and establishing a foundation for future explorations into diverse cognitive and clinical domains.

### 2.2. Introducing the Thoughtseeds Framework

**Figure 1:**
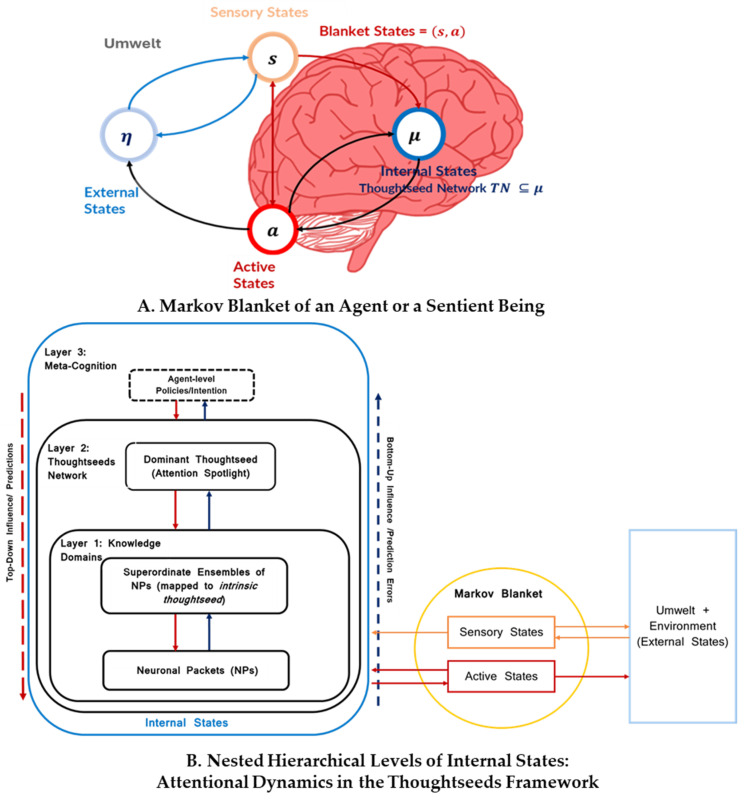
**Hierarchical Thoughtseed Framework within a sentient being.** (**A**) Partitioning of an agent’s internal states and external states through the Markov blanket (Ref [34]). The Markov blanket, comprising sensory (**s**) and active states (**a**), mediates the interaction between internal states internal states (**μ**), and external states (**η**). Internal states can only influence external states through active states, while external states can only influence internal states through sensory states. This separation allows the internal states, which house the Thoughtseed Network (**TN**), to operate with a degree of autonomy, generating predictions and selecting actions based on its internal model of the world. (**B**) The **nested hierarchical organization** of attentional processes within the brain’s internal states, as detailed in Figure 1A. It illustrates three levels—**knowledge domains (KDs)**, the **Thoughtseed Network (TN)**, and **meta-cognition**—each enclosed in its own Markov blanket, forming a hierarchical structure rooted in active inference and Global Workspace Theory (GWT). **Knowledge domains (KDs):** The base level comprises self-organizing units of embodied knowledge. Each KD has a Markov blanket that interfaces with sensory inputs and actions, providing a neuronal foundation for both conscious and unconscious processing. **Thoughtseed Network (TN):** The TN represents interactions among **thoughtseeds**—*attentional agents*—competing for dominance in the Global Workspace via winner-takes-all dynamics, shaping the content of conscious experience. **Meta-cognition:** The meta-cognitive agent oversees the Thoughtseed Network via attentional precision, and meta-awareness monitors a detection system so that the behavior is aligned with intentionality, guided by global goals and policies. **Bidirectional information flow:** Blue arrows indicate bottom-up processing (e.g., prediction errors), whereas red arrows indicate top-down processing (e.g., predictions). This bidirectional flow reflects the dynamic interaction across cognitive levels, where each layer functions as a Markov blanket, facilitating selective information exchange. **Markov Blanket interactions:** At the system’s boundary, the Markov blanket interfaces with the external states, labeled “Umwelt + Environment,” through sensory states and active states. This interaction aligns with embodied cognition’s emphasis on the sentient being’s engagement with its surroundings, shaping cognitive processes through sensory inputs and active outputs.

The Thoughtseed Framework (see Figure 1) proposes a hierarchical, agent-based modeling of cognitive processes in order to represent how the brain manages complex functions such as decision-making, problem-solving, and planning. Rooted in the *Global Workspace Theory (GWT)* [44,45,46,47] and principles of *embodied cognition* [1,49], the framework illustrates how conscious experience may emerge from the dynamic interplay of internal cognitive processes, the body, and the external environment.

**Figure 2:**
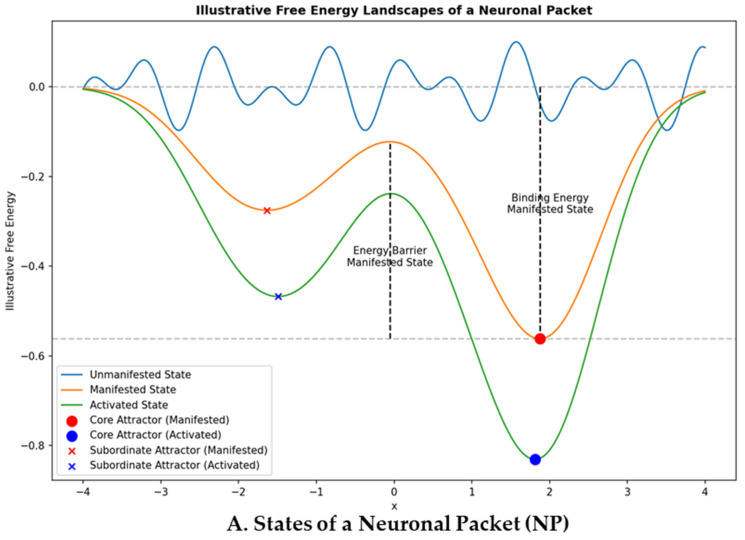

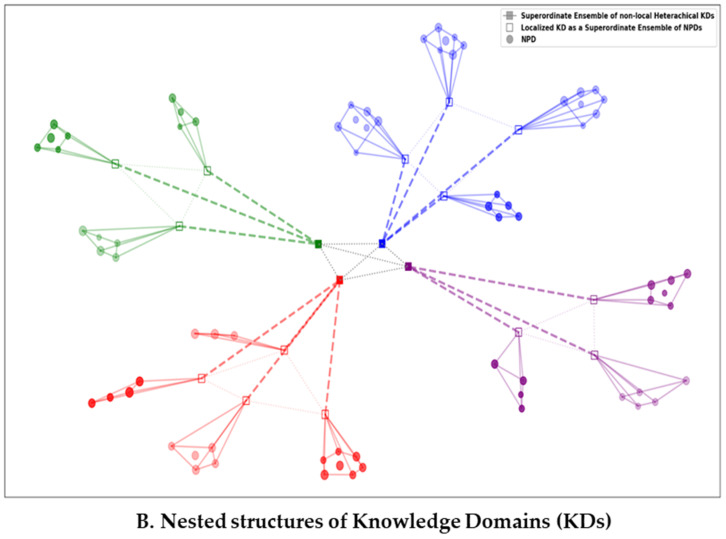
**Neuronal packets and knowledge domains.** (**A**) States of a neuronal packet (NP), showing how it transitions from an unmanifested to a manifested state, forming a Markov blanket that encapsulates its knowledge structure. In its activated state, the NP processes sensory inputs and generates actions. (**B**) Multiple NPs organize into knowledge domains (KDs), integrating sensory data and actions into a hierarchical and heterarchical framework (Level 1 in Thoughtseed Framework). The responses from NPs’ activated states feed into KDs, enabling thoughtseed dynamics and competition in the Global Workspace, ultimately shaping conscious experience. (**A**) This figure illustrates the states of a neuronal packet (NP) using a free energy landscape, with the *x*-axis representing internal states and the *y*-axis indicating free energy. The NP exists in three states: **Unmanifested State:** A potential neural configuration shaped by evolutionary priors, shown as a shallow local minimum (blue curve). **Manifested State:** Forms after repeated stimulus exposure, leading to a phase transition and the formation of a stable Markov Blanket—with its encapsulated knowledge structure. It includes a core attractor (red dot) as the primary neural pattern and subordinate attractors (red ‘x’) as secondary patterns. The vertical dashed line marks the energy barrier, and binding energy is the distance from the core attractor to the zero-free energy level (horizontal dashed line). **Activated (or Spiking) State:** A transient state characterized by heightened neural activity within the manifested NP ensemble, triggered by the dominant thoughtseed and generating a response influencing behavior or cognition (green curve). (**B**) KDs are shown as colored squares, each representing specialized knowledge areas: Unfilled squares indicate localized KDs (superordinate ensembles of NPs, or neuronal packet domains, NPDs). A filled square represents a higher-order, heterarchical KD integrating knowledge across domains. **Organization:** The arrangement of NPs within or connected to KDs visually represents the *hierarchical* nature of knowledge representation. The higher-order KD suggests a *heterarchical* organization that allows for a more complex integration of knowledge across domains. **Dynamic Interplay:** Connections between NPs and KDs represent the flow of information and influence. NPs provide raw data, while KDs interpret and contextualize this information, contributing to *thoughtseed emergence* from the dynamic interplay of information processing within and between KDs.

At its core, the Thoughtseed Framework revolves around three key concepts:

- **Knowledge domains** (KDs): Organized units of knowledge within the brain that serve as the *structural basis for thought* (as a thoughtseed can be associated with specific knowledge structures, which function as its core attractor, along with secondary attractors that can form nested hierarchies across scales, as discussed in Figure 2B).
- **Thoughtseeds:** *Attentional agents* that interact within a Thoughtseed Network, enabling the framework to model the *emergence, evolution, and shifting of thoughts*—particularly during meditation and mind-wandering. Thoughtseeds operate within the Global Workspace, where a *dominant thoughtseed* emerges through a *winner-takes-all* dynamic.
- **Meta-cognition:** It monitors the Thoughtseed Network (and the Global Workspace), aligning the sentient being with its current intentionality, goals, and policies. At the top level of the nested hierarchy, it acts as an irreducible Markov blanket [22,50,51], separating its internal processes from the lower-level cognitive dynamics, which are themselves separated from external influences by the agent-level Markov blanket.

For key concepts of Thoughtseeds Framework, see Table 2.

### 2.3. Knowledge Domains (KDs)

Knowledge domains (KDs) are self-organizing units of embodied knowledge within the Thoughtseed Framework, resembling recurring, metastable brain states. These units encapsulate structured knowledge derived from underlying neuronal packets (NPs) or their superordinate ensembles, as depicted in Figure 2. Each KD represents a distinct area of expertise and serves as a nested knowledge repository [52] within the brain’s internal states, integrating sensory inputs, retrieved memories, beliefs, experiences, policies, emotions, and learned patterns. KDs can vary in scope, from *local* domains representing specific knowledge or skills to *non-local* domains that integrate information across multiple areas. They exhibit both *hierarchical* [22,53] and *heterarchical* structures [54], enabling flexible and context-dependent knowledge retrieval for adaptive behavior. Context-dependent binding [55,56] within KDs integrates information across scales, forming coherent percepts in the Global Workspace.

KDs possess an affective dimension that reflects the emotional valence and arousal associated with their content, shaping subjective experiences and influencing behavior and decision-making processes [57]. This emotional component is vital in guiding behavior, although certain KDs related to abstract knowledge, such as mathematical rules, may lack affective content.

KDs can be broadly categorized into two types, similar to the typical method of classifying memories [58].

- **Procedural KDs:** These domains encode learned skills, motor control, and sensorimotor processes, guiding automatic behaviors without requiring conscious thought. For instance, riding a bicycle relies on a procedural KD, which is reflected in the influence of the active states on the environment.
- **Declarative KDs:** These domains store and retrieve explicit knowledge, such as facts, events, and conscious memories, which recent studies suggest may be organized through grid-like coding mechanisms [30], providing the foundation for abstract reasoning and conscious content in the Global Workspace.

The internal states in Figure 1 at Level 1 include both procedural and declarative KDs, whose behavior changes over time with expertise.

### 2.4. Thoughtseeds Network

Thoughtseeds are dynamic cognitive units intrinsic to stable knowledge domains (KDs), which are superordinate ensembles of neuronal packets (NPs) [32,33,34]. KDs require repeated learning and consolidation to stabilize, enabling distinct cognitive representations to emerge. Once formed, thoughtseeds exhibit robust attractor dynamics, with a stable core attractor (the KD’s dominant theme) and flexible subordinate attractors for contextual adaptation [59]. Empirical evidence from meditation research highlights the hippocampus’s role in the spontaneous arising of thoughts [60], supporting the dynamic nature of these cognitive units. Thoughtseeds then compete via a winner-takes-all mechanism [46] to gain dominance in the Global Workspace, and guide, action, and decision-making, gaining the *attention spotlight* [61].

Thoughtseeds enhance a system’s agency through active inference, generating predictions, influencing actions, and updating internal models based on sensory feedback [62]. Thus, KDs serve as abstract spaces where thoughtseeds form complex representations and narratives, reflecting how intelligence generalizes from navigating physical spaces to abstract spaces for higher-level cognition [63].

#### 2.4.1. Thoughtseed States

Thoughtseeds are assumed to be dynamic cognitive units intrinsic to stable knowledge domains (KDs), which are superordinate ensembles of neuronal packets (NPs) consolidated through repeated learning. The states of thoughtseeds—unmanifested, manifested (with inactive and active sub-states), and dominant—reflect the stability and coherence of their associated KDs, illustrating their progression from latent potential to orchestrating influence on conscious experience.

##### Unmanifested State

Initially, thoughtseeds exist in an unmanifested or latent state representing an emerging pattern of neural activity shaped by evolutionary priors but not yet stable enough for activation. It corresponds to a shallow local minimum in the free energy landscape, where the thoughtseed remains dormant until the KD achieves sufficient coherence. This aligns with the “unmanifested state” depicted in the diagrams (Figure 2A).

##### Manifested State

Once a KD stabilizes through repeated learning, the corresponding thoughtseed transitions to a manifested state. In this state, it possesses a well-formed core attractor—reflecting the KD’s stable thematic content—and flexible subordinate attractors that enable contextual adaptation. The manifested state is subdivided into two sub-states:

- **Inactive:** The thoughtseed is present within the stabilized KD but does not actively influence conscious processes. It exists as a stable neural pattern that is primed for potential activation.
- **Active:** The thoughtseed engages in cognitive processing (part of the active thoughtseeds pool), contributing to perception and action, though it does not yet dominate the Global Workspace.

##### Dominant State (Activated/Spiking State)

Through competitive interactions governed by a winner-takes-all mechanism, a thoughtseed may achieve a dominant state, also referred to as an activated or spiking state. In this state, it minimizes the *cumulative Expected Free Energy (EFE)* [64]—optimizing prediction accuracy while managing computational resources—and enters the Global Workspace to drive conscious experience, guiding attention, perception, and decision-making. The dominant thoughtseed functions as a *pullback attractor* [65], orchestrating information across KDs to form coherent representations. This process establishes a transient Markov Blanket, as depicted in the diagrams (Figure 1B, Level 2), and gains the attention spotlight, which maintains the system’s autonomy and computational independence by mediating interactions between internal states and the external environment (Umwelt) [66].

#### 2.4.2. Thoughtseed Definition

Here, a thoughtseed is defined as a transient, higher-order cognitive unit that emerges from the coordinated activity of neuronal ensembles within a Thoughtseed Network, encapsulated by a Markov-blanketed structure. Functioning as an *attentional agent*, a thoughtseed integrates and propagates information across knowledge domains (KDs), structured repositories of knowledge derived from neuronal packets.

Thoughtseeds exhibit attractor dynamics, with a core attractor representing their most stable configuration and subordinate attractors providing contextual flexibility. Within a dynamic cognitive landscape, thoughtseeds compete for prominence via a *winner-takes-all mechanism* [46]—allowing the *dominant thoughtseed* to enter the Global Workspace, gaining the *attention spotlight* [61] which shapes conscious experience and guides perception, action, and decision-making.

Thereby, thoughtseeds act as *minimizers of Expected Free Energy (EFE)*, optimizing internal models and driving inference within a network of interacting dynamical attractors. Through this process, they enable adaptive cognition, balancing epistemic (information-seeking) and pragmatic (goal-directed) affordances while continuously reshaping the agent’s engagement with its Umwelt/subjective environment [67].

Even in the absence of direct sensory input, thoughtseeds can re-emerge due to intrinsic dynamics, contributing to *phenomena such as mind-wandering and spontaneous cognition* [68,69].

### 2.5. Meta-Cognition

Meta-cognition operates as a dynamic monitoring system within this framework, continuously assessing and adjusting the sentient being’s overarching policies and goals to align with internal intentions and external demands. It oversees the competition among thoughtseeds in the active pool, depicted as Layer 2, through two primary mechanisms:

- **Attentional Precision:** By modulating the precision of specific thoughtseeds, meta-cognition enhances or suppresses sensitivity to sensory evidence, prioritizing those most relevant for conscious access [70]. This process, shown as blue arrows from agent-level policies/intentions to the Thoughtseed Network (especially the dominant thoughtseed), sharpens perception based on current goals and context.
- **Meta-Awareness:** This mechanism detects shifts in behavior that do not correspond to global policies and triggers corrective actions of potential actions within the Thoughtseed Network [71]. Feedback loops (red arrows) enable the system to reflect on its strategies and adapt dynamically.

Thoughtseeds, embodying policies, goals, and affordances, steer adaptive behavior by shaping perception and action. Meta-cognition guides this selection, aligning it with a hierarchical policy structure where thoughtseeds at varying abstraction levels contribute to execution [15,64,72,73].

## 3. Applying Thoughtseeds Framework to Focused Attention Meditation Simulation

### 3.1. Overview

Inspired by previous work in *“computational phenomenology”* [74], we developed a three-level hierarchical model of focused-attention Vipassana meditation [37,38]. We further leveraged the concepts of meta-awareness modulation and attentional precision from the meta-cognition layer, along with concepts related to attentional fatigue and meta-awareness charge [74]. The framework implements an agent-based model in which meditation states emerge from interactions across multiple levels of organization, rather than following predetermined transitions. It is grounded in the principles of the Global Workspace Theory (GWT) [44,45,46,47].

**Level 1: Knowledge Domain** (mapped to its *intrinsic thoughtseed*)**:** The foundational level represents neurobiological substrates as knowledge domains—specialized neural ensembles that encode fundamental cognitive functions. In this simulation, we established a one-to-one correspondence between the knowledge domain and its intrinsic thoughtseed, which in reality can be significantly more complex. In this simulation, Level 1 maps to the activation levels of a thoughtseed.

**Level 2: Thoughtseed Network:** Thoughtseeds emerge at an intermediate level as attentional attractors arising from coordinated activation patterns across knowledge domains. The Thoughtseed Network implements interactions extracted through lagged correlation analysis, where five thoughtseeds (‘breath_focus’, ‘equanimity’, ‘pain_discomfort’, ‘pending_tasks’, ’self_reflection’) compete and cooperate through mutual excitation and inhibition, analogous to competitive neural assemblies in theoretical neuroscience models [75,76].

**Level 3: Meta-Cognition:** At the highest level, a meta-awareness system monitors the Thoughtseed Network and regulates state transitions through the following:

- State-dependent awareness levels that modulate thoughtseed interactions;
- Probabilistic detection mechanisms for mind-wandering that improve with expertise;
- Regulatory interventions that implement attentional control processes.

This system implements key aspects of neurocognitive theories of mindfulness [74,77], particularly regarding the development of meta-awareness as a distinct regulatory function in meditation training.

### 3.2. Meditative States and Empirical Grounding

**Figure 3:**
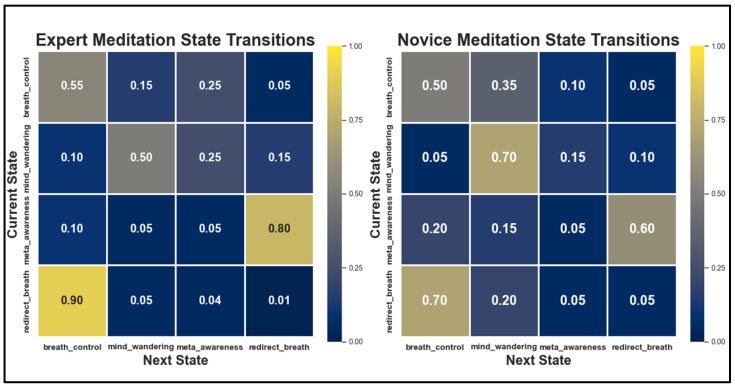
**State transition matrices derived from empirical research [37,38].** For experts (left panel), in the ‘breath_control’ state, practitioners maintain focus 55% of the time and engage in meta-awareness 25% of the time, reflecting adaptive attentional dynamics [36,78]. In the ‘mind_wandering’ state, experts exhibit self-regulation, spending 50% of the time in this state with a 25% probability of transitioning to ‘meta_awareness’, indicating timely recognition of competing thoughts. The ‘meta_awareness’ state transitions to ‘redirect_breath’ with an 80% probability, which then returns to ‘breath_control’ 90% of the time [38]. For novices (right panel), ‘breath_control’ shows reduced stability with focus maintained 50% of the time and a 35% transition to ‘mind_wandering’. In the ‘mind_wandering’ state, novices remain 70% of the time, while ‘meta_awareness’ transitions to ‘redirect_breath’ 65% of the time. The ‘redirect_breath’ state returns to ‘breath_control’ 70% of the time but lapses to ‘mind_wandering’ 20%, indicating weaker attentional control [79,80]. These matrices, derived from focused attention meditation research [78,79,80], serve as a reference for understanding the 4 state cyclical patterns [37,38] in Vipassana meditation practice.

Each meditation state (breath_control, mind_wandering, meta_awareness, redirect_breath) forms a distinct attractor basin in the activation space of the Thoughtseed Network, guided by thoughtseed dynamics and empirical biases rather than being explicitly programmed. These states align with the four-state cycle identified in Vipassana meditation research [37,38] (see Figure 3).

Thoughtseeds compete for access to the Global Workspace via winner-takes-all dynamics [46]. For instance, breath_control prioritizes breath_focus as the dominant attentional agent, whereas mind_wandering sees competition among pain_discomfort and pending_tasks. Meta_awareness and redirect_breath elevate self_reflection and equanimity as key attentional units.

### 3.3. Learning Framework: Rule-Based Optimization

In the context of agent-based modeling of thoughtseed dynamics during Vipassana meditation, we have developed a rule-based hybrid learning framework. This framework optimizes weights and interaction patterns through rule-based constraints, thereby obviating the necessity of manual parameter adjustment. During the training phase, state-thoughtseed associations are established, resulting in emergent transition patterns that align with empirical meditation research [38,78].

#### 3.3.1. Mathematical Framework for Learning

Our hybrid framework models Vipassana meditation as a dynamic, nonlinear system in which attentional states arise from competing cognitive processes. Instead of relying on predetermined sequences, the model generates naturalistic transitions between meditation states through activation-based competition and threshold dynamics, detailed in the Supplementary Materials.

##### Attractor-Based Weight Matrix Construction

**Figure 4:**
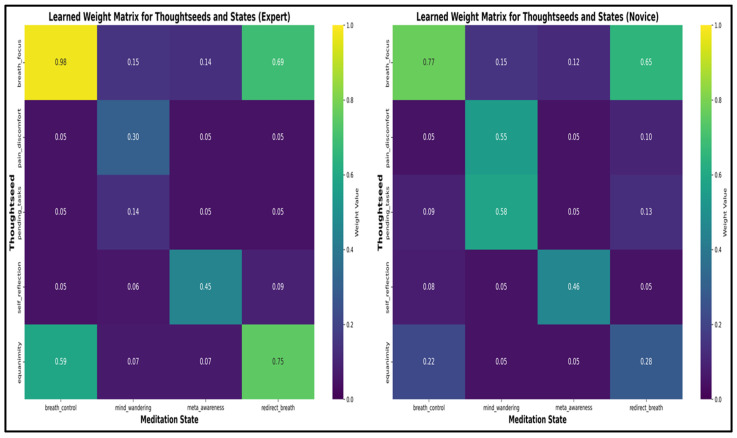
**Learned weight matrices after learning.** The matrices (**left**: **expert**, **right**: **novice**) reveal experience-dependent differences: **Attentional Focus Enhancement:** Experts display stronger breath_focus activation during breath_control (0.98 vs. 0.78 in novices), reflecting enhanced executive control [79]. **Distraction Reduction:** Novices show higher distraction thoughtseed activation in mind_wandering (e.g., pain_discomfort: 0.55, pending_tasks: 0.58) compared to experts (0.30, 0.14), consistent with reduced default mode network activity in experienced meditators [78]. **Equanimity Development:** Experts exhibit stronger equanimity weights, especially during redirect_breath (0.75 vs. 0.28 in novices), supporting improved emotional regulation [81]. **Meta-cognitive Awareness and Redirect Breath:** Both groups show similar self_reflection activation during meta_awareness (experts: 0.45, novices: 0.46), but experts show greater redirecting attention to breath capabilities (experts: 0.75, novices: 0.28). **Neural Pattern Consistency:** Experts’ tighter weight clustering in adaptive states (breath_control, redirect_breath) indicates consistent neural recruitment, reflecting neuroplasticity from long-term practice [82,83].

The framework defines a weight matrix **W**, which defines distinct attractor landscapes of thoughtseeds for each meditation state (see Figure 4).


(1) $$W_{ts,state}=\left\{\begin{array}{c} \omega_{base}\cdot U\left(0.9,1.1\right),\;if\;ts\;\in primary\_attractor\;\\ \omega_{base}\cdot U\left(0.7,0.9\right),\;if\;ts\;\in secondary\_attractors\\ \omega_{base}\cdot U\left(0.05,0.2\right),\;otherwise \end{array}\right\}$$

This matrix assigns higher weights to thoughtseeds most relevant to each state—like breath_focus for breath_control—using predefined rules based on empirical meditation data, creating stable patterns or “attractors” that guide attention. Over time, these weights shape how strongly each thoughtseed influences a state, reflecting expertise-driven differences in focus and distraction management.

These normalized weight matrices quantify how meditation expertise optimizes attentional resource allocation, with experts enhancing focus while reducing distractions—a configuration supporting sustained present-moment awareness.

##### Nonlinear Thoughtseed Activation Dynamics

Thoughtseed activation evolves through a complex interplay of nonlinear mechanisms that govern their competitive interactions within the Thoughtseed Network [84,85,86,87]. This nonlinearity means small changes in one thoughtseed’s activation—like a spike in pain_discomfort—can amplify or suppress others, mimicking the dynamic shifts seen in real meditation. Such interactions allow the system to oscillate naturally between focus and distraction, reflecting the brain’s adaptive responses. It captures the shifting attentional states observed in Vipassana meditation by modeling three key processes:

(1) **Distraction growth,** where distracting thoughtseeds (e.g., pain_discomfort, pending_tasks) exhibit spontaneous activation spikes, reflecting the neural underpinnings of mind-wandering [68,69];

(2) **State-specific modulation,** such as during mind-wandering when low meta-awareness strongly suppresses breath_focus (e.g., by a factor of 0.05) while enhancing distractions (e.g., by a factor of 1.2), aligning with findings on attentional lapses in novices [36,88];

(3) **Feedback-driven interactions,** which enable thoughtseeds to influence each other within the network, mirroring the recurrent neural dynamics seen in sustained meditation [84,85].

These mechanisms, detailed in the Supplementary Materials, collectively model the multistable attentional dynamics characteristic of meditation, in which focused attention oscillates with spontaneous thought [68,69].


(2) $$\alpha_{t+1}=\left(1-\gamma \right)\cdot \alpha_{t}+\gamma \cdot \alpha_{t}^{target}+\Delta \alpha_{t}^{dist}+\eta_{t}$$

##### Meta-Awareness Regulation with State-Dependent Dynamics

Meta-awareness $\mu_{t}$ evolves according to state-dependent dynamics:


(3) $$\mu_{t}=\left\{\begin{array}{c} max\left(0.55,0.6-0.05+\epsilon_{t}\right),if\;state=mind\_wandering\;and\;dominant\_ts\in distractions\\ max\left(0.55,0.6+0.4+\epsilon_{t}\right),if\;state=mind\_wandering\;and\;dominant\_ts\notin distractions\\ min\left(0.85,0.6+0.25+\epsilon_{t}\right),if\;state=redirect\_breath\\ min\left(0.75,0.6+0.2+\epsilon_{t}\right),if\;state=breath\_control\\ min\left(0.9,0.6+0.3+\epsilon_{t}\right),if\;state=meta\_awareness \end{array}\right\}$$

**Figure 5:**
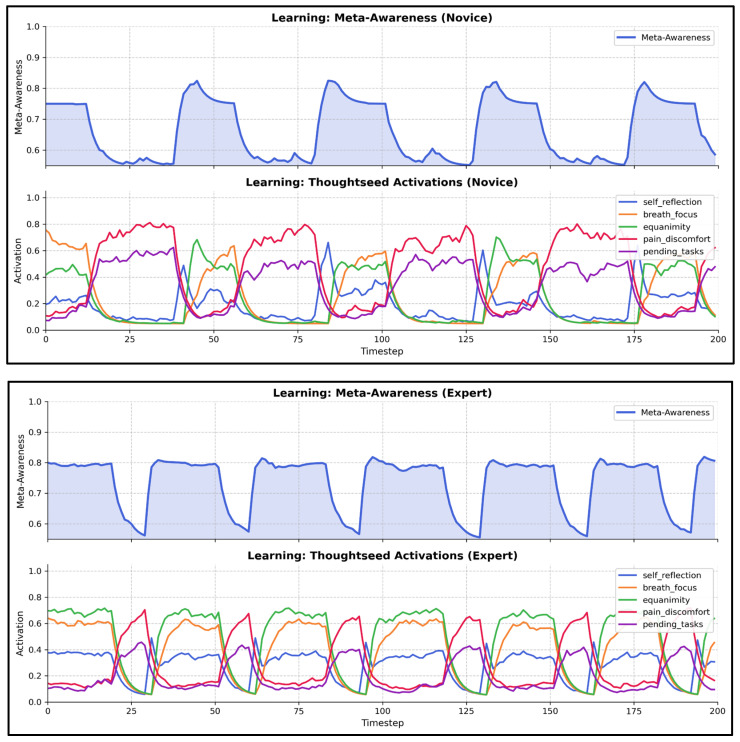
**Thoughtseed activations and meta-awareness during learning.** This composite plot shows the activation trajectories of five thoughtseeds (‘self-reflection’, ‘breath_focus’, ‘equanimity’, ‘pain_discomfort’, ‘pending_tasks’) along with meta-awareness for **novice** meditators (**top panel**) and **expert** meditators (**bottom panel**) during the learning process.

Figure 5 shows trajectories of activation levels of individual thoughtseeds and meta-cognition. Awareness levels are dynamically adjusted based on the current meditative state and the dominant thoughtseed, such as slightly decreasing during mind_wandering with distractions (e.g., pending_tasks) or significantly increasing in meta_awareness, guided by expertise-driven thresholds. Random noise adds variability, reflecting natural fluctuations.

##### Statistical Learning of Transition Patterns

The model builds a transition probability matrix from observed transitions, starting without a fixed **T** matrix and using initial thresholds to guide shifts based on thoughtseed activations and meta-awareness. During learning, it dynamically tracks and counts state transitions (e.g., breath_control to mind_wandering), normalizing these frequencies at the end to form the T matrix, reflecting emergent patterns like focus-distraction oscillations [36,38,89]. This process learns probabilities from state-specific dynamics, meta-awareness, and natural/forced transitions, adapting over time without explicit optimization.

(4) $$T_{i,j}=\frac{count\left({State}_{i}\to {State}_{j}\right)}{total\_transitions\_from\_{State}_{i}}$$

It also captures mean activation patterns at transition points.

(5) $${\bar{\alpha }}_{i,j}^{trans}=\frac{1}{n_{i,j}}\sum_{k=1}^{n_{i,j}}\alpha_{i\to j}^{\left(k\right)}\;\;$$

##### Threshold-Based State Transitions

State transitions arise from threshold-crossing events in thoughtseed activations:


(6) $$P\left({State}_{t}\to {State}_{t+1}\right)=\left\{\begin{array}{c} 1,if\phi \left({State}_{t}\alpha_{t}\right)>\theta_{transition}\;and\;{dwell}_{t}\geq {dwell}_{min}\\ 0,\;otherwise \end{array}\right\}$$

Transitions occur in either of the following forms:

- **Natural transition:** Activation pattern exceeds threshold (captured in transition_activations);
- **Forced transition:** Dwell time limit reached without natural transition.

**Figure 6:**
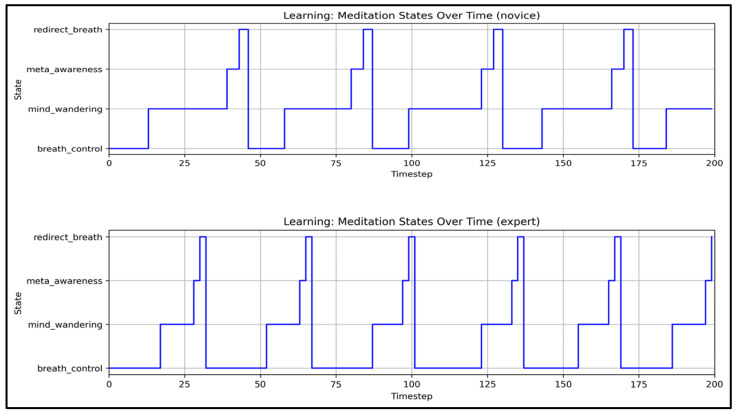
**Mediation states’ evolution during learning.** These plots illustrate the temporal progression of meditation states—breath_control, mind_wandering, meta-awareness, and redirect_breath—over 200 timesteps for **novice** (**top panel**) and **expert** (**bottom panel**) meditators. State transitions, constrained by dwell time limits (mean ± 2 SD) and modulated by meta-awareness (0.55–0.9), demonstrate a 4-state cyclical model of focused attention in Vipassana meditation [36,38].

These threshold mechanisms model attractor transitions observed in neural recordings during shifting cognitive states [39] and implement phase transitions consistent with multistable attentional dynamics [85,86,90]. Figure 6 shows how mediation states evolve during learning.

#### 3.3.2. Learning Results

The rule-based hybrid learning framework produces a weight matrix encoding relationships between thoughtseeds and meditative states, self-organizing into stable state transitions over 200 timesteps without manual tuning. Thoughtseeds are modeled as competitive attentional agents, yielding emergent patterns that align with empirical meditation research [36,37,38,79].

##### Emergent Patterns and Experience-Dependent Variations

The learning patterns reveal significant differences between novice and expert meditators:

- **Attention Stabilization**: Experts maintain breath_focus activation during breath_control periods within a stable range of (0.50–0.60) with minimal fluctuations. In contrast, novices exhibit greater variability (0.30–0.50), alongside frequent intrusions from distracting thoughtseeds (e.g., pain_discomfort, pending_tasks), indicative of weaker attentional control [36,38].
- **State Transition Efficiency**: Experts demonstrate shorter mind_wandering episodes (8–12 s vs. 20–30 s for novices). They also recover more efficiently through meta_awareness and redirection states, reflecting enhanced self-monitoring and the ability to redirect focus. These patterns align with EEG findings showing reduced default mode network activity in experienced meditators [38,91].
- **Meta-Awareness Dynamics**: Experts maintain higher meta-awareness levels (0.75–0.9) with less variance than novices (0.6–0.8, with greater fluctuation), aligning with neuroimaging evidence of enhanced prefrontal monitoring in experienced meditators [36].

##### Alignment with Computational Models

Our approach aligns with computational models of mind-wandering [88], which simulated mind-wandering as shifts between on-task and off-task states using reinforcement learning to model attentional dynamics influenced by meta-awareness. We extend their framework by incorporating empirically derived transition probabilities that reflect expertise-related differences in meditation states [39,79]. This statistical framework captures both moment-to-moment variability in meditation (e.g., oscillation between focused attention and mind-wandering) and long-term patterns emerging with practice (e.g., increased attentional stability).

The learning approach minimizes prediction error through three rule-based mechanisms: (1) gradual momentum-based updates (90/10 blending), reflecting neural population inertia; (2) state-specific suppression and facilitation effects, implementing attentional competition observed in meditation neuroscience; and (3) bounded noise levels (novice: 0.08, expert: 0.04), modeling precision improvements with practice. This hybrid framework—combining rule-based bootstrapping with emergent dynamical models—captures the phenomenology of meditative attention through implicit learning, mirroring the adaptive processes observed in long-term meditation practice [79].

## 4. Simulation Results

We developed an agent-based computational model of thoughtseeds as attentional units during the Vipassana meditation. This simulation implements a multi-level dynamical system in which meditation states emerge from lower-level interactions between cognitive elements, rather than following predetermined transitions. The three-level hierarchical model is grounded in neurocognitive theories of meditation [36,37,38].

During the simulation, the components generated realistic meditation trajectories with expertise-dependent differences in attentional stability, distraction vulnerability, and meta-cognitive efficiency. Unlike traditional rule-based models, state transitions emerge naturally when activation thresholds are crossed, creating rule-based patterns that match empirical observations without hard-coding the meditation cycle.

### 4.1. Thoughtseed Interaction Network: The Foundation of Emergent Dynamics

The interaction network encodes how thoughtseeds influence each other through a **data-driven Granger causality framework** [92,93]. This approach captures directional influences by analyzing how one thoughtseed’s activation patterns predict another’s future states, distinguishing genuine causal relationships from mere correlations.

The extraction methodology implements a three-stage process:

**Stage 1: Granger Causality Testing**

For each thoughtseed pair (*x*, *y*), we test whether past values of *x* help predict future values of *y* beyond what *y*’s past values alone can predict:

(7) $$G_{x\to y}=\sum_{l=1}^{L}\frac{RSS\left(y|y_{past}\right)-RSS\left(y|y_{past},x_{past}\right)}{RSS\left(y|y_{past},x_{past}\right)}\;$$

where *RSS* represents the residual sum of squares from the regression models, and represents the maximum lag (five timesteps).

**Stage 2: Statistical Significance and Strength Calculation**

Statistical significance was determined using chi-squared tests, with causal strength inversely proportional to *p*-values. This approach ensures that only statistically significant relationships (*p* < 0.05) are identified as causal, with strength reflecting the confidence in the causal relationship.

**Stage 3: Calibration and Constraints**

The final interaction weights were scaled and bounded to ranges (−0.7, 0.7). This formulation combines statistical rigor with constraints and filters weak connections (threshold < 0.1) while preserving significant causal relationships.

**Weighted Causal Influence**


(8) $$W_{i,j}=0.7\;\cdot Causal\;Weights+0.3\;\cdot Baseline\;Correlations$$

**Figure 7:**
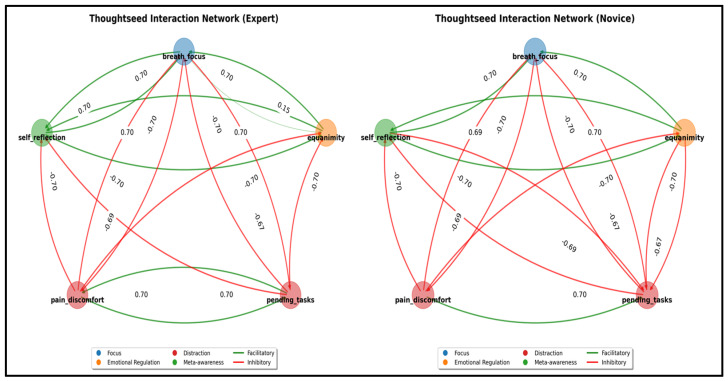
**Thoughtseed interaction network.** These thoughtseed interactions differ between meditation experience levels (**novice** in **right panel** and **expert** in **left panel**), where red represents inhibitory connections and green represents facilitatory connections. The data-driven analysis reveals several key differences: **Enhanced Meta-Cognitive Processing:** Experts show stronger facilitatory connections from breath focus to self-reflection (+0.70 vs. novices’ +0.00), indicating that sustained attention to breath becomes integrated with meta-cognitive awareness through practice [36]. **Refined Distraction Management:** Novices show mutual reinforcement between distraction types (pain_discomfort → pending_tasks: +0.70), while experts demonstrate no such reinforcement (+0.00), indicating better separation between different distraction categories. **Improved Breath-related Regulation:** Experts develop more targeted inhibitory control (breath_focus → pending_tasks: −0.69) while simultaneously reducing inhibition toward sensations (breath_focus → pain_discomfort: +0.00 vs. novices’ −0.70). **Equanimity Cultivation:** Experts show a positive connection from breath_focus to equanimity (+0.25) that is absent in novices, supporting theoretical accounts of breath awareness fostering equanimity with sustained practice [80]. **Reduced Negative Interference:** Both self_reflection and equanimity show less inhibitory relationships with pending_tasks in experts, suggesting more balanced integration of attention networks rather than oppositional relationships.

This weighted approach prioritizes differential effects (70%) over basic correlations (30%), identifying genuine causal relationships while filtering out spurious connections. The resulting weights are thresholded |W| > 0.15 and scaled to a plausible range (−0.6 to 0.6) (see Figure 7).

The expert matrix reveals a more balanced network with enhanced regulatory pathways between focus-related thoughtseeds and stronger inhibitory control over distractions—quantifying how meditation training restructures attentional dynamics through neuroplasticity.

### 4.2. Multi-Scale Dynamical System for Meditation Simulation

Meditation simulation implements a hierarchical dynamical system in which thoughtseed interactions give rise to meditation states through emergent properties, mirroring how neural dynamics generate cognitive states in the brain [48,84]. This three-level architecture captures the emergence of meditation states from coupled neuro-dynamical processes operating at different timescales, consistent with models of metastability where transient neural synchronization drives cognitive state transitions [38,39,90]. Both the *bottom-up emergence of meditation states* and *top-down modulation* via *meta-cognitive processes* are facilitated, reflecting the dynamic interplay observed in mindfulness practices [74].

#### 4.2.1. Individual Thoughtseed Dynamics


(9) $$\alpha_{i}(t+1)=r_{i}\cdot {Target}_{i}\left(t\right)+\left(1-r_{i}\right)\cdot \alpha_{i}(t)$$

Momentum-based update equations govern each thoughtseed’s activation dynamics, balancing incoming influence with neural inertia. The responsiveness parameter reflects expertise-dependent neuroplasticity, with experts exhibiting higher stabilizing values (0.7–0.8) than novices (0.6–0.7). This aligns with findings that meditation expertise enhances attentional stability by strengthening neural circuits involved in sustained focus [79,94]. These dynamics lay the foundation for thoughtseed interactions within a broader network, thus influencing meditative state transitions.

#### 4.2.2. Thoughtseed Network Dynamics


(10) $${Target}_{i}\left(t\right)=W_{i}^{s}+\sum_{j\neq i}W_{ij}\cdot \alpha_{j\left(t\right)}\cdot \tau +\gamma_{i}^{s}\cdot \tau +m\left(t\right)\cdot \beta_{i}$$

At the network level, a modified Wilson–Cowan-type model simulates competing thoughtseed populations, where facilitatory and inhibitory connections shape their interactions. State-specific terms modulate baseline activation tendencies, while meta-awareness provides top-down regulation, akin to the frontoparietal attentional control observed in meditation studies [95,96]. This creates attractor basins corresponding to distinct meditation states, such as focused attention or mind-wandering, enabling the system to oscillate between these states [36].

#### 4.2.3. Emergent State Transition Dynamics


(11) $$P\left(State_{t+1}=j\;|State_{t}=i,\alpha_{k\left(t\right)}\right)=f\left(T_{ij},\alpha_{k\left(t\right)},\theta_{k}\right)$$

State transitions arise from a hybrid process in which accumulated thoughtseed activations trigger shifts between meditation states, such as from focused attention to mind-wandering. This cyclic pattern mirrors real-time meditation sampling studies that show characteristic oscillations in attentional focus [39]. Meditation expertise enhances transition efficiency, with experts exhibiting longer focused periods and faster recovery from mind-wandering episodes, reflecting improved neural synchronization and attentional control [89,97].

#### 4.2.4. Dominant Thoughtseed Dynamics in the Hierarchical Framework

The hierarchical structure facilitates “winner-takes-all” dynamics [46], where the thoughtseed with the highest activation becomes dominant at each timestep, influencing both state transitions and meta-awareness:


(12) $$\tau^{*}={argmax}_{\tau_{i\epsilon Τ}}\alpha \left(\tau_{i},t\right)$$

When a distraction thoughtseed (e.g., pain_discomfort) exceeds its activation threshold, it increases the likelihood of transitioning to a mind-wandering state, whereas a dominant self-reflection thoughtseed can rapidly elevate meta-awareness, shifting focus back to meditative states. Meta-awareness responds dynamically to these shifts, remaining low during distraction dominance but rising when ‘self_reflection’ prevails, reflecting frontoparietal network modulation [95]. Additionally, the dominant thoughtseed synchronizes network activity, creating transient attractor states like redirect_breath that mirror neural synchronization patterns in meditation [83]. Detailed equations governing these interactions are provided in the Supplementary Materials.

**Figure 8:**
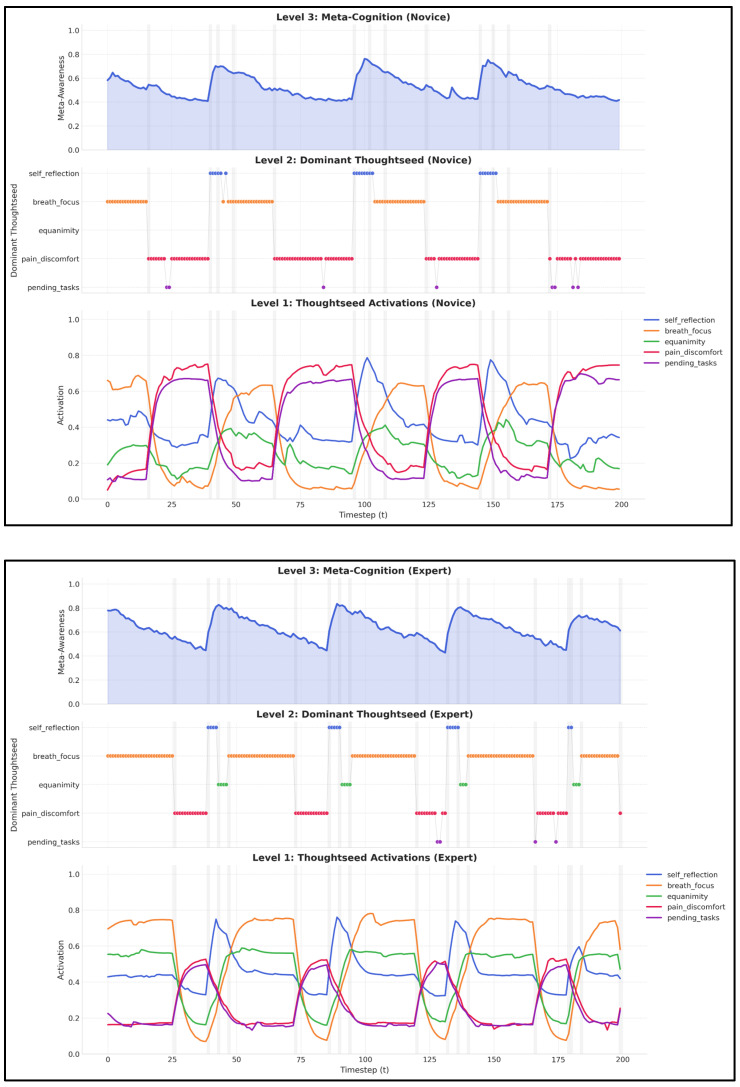
**Hierarchical organization of meditation dynamics.** This figure illustrates a three-level organization of meditation dynamics for **novice** (**top panel**) and **expert** (**bottom panel**) meditators: It demonstrates how meditation emerges from three interconnected levels: **Level 1—Thoughtseed Activations:** Competitive dynamics between five color-coded thoughtseeds (e.g., breath_focus, self_reflection), with continuous activation trajectories showing the evolution of attention, distraction, and meta-cognitive processes over time. **Level 2—Dominant Thoughtseed** (**middle**): Discrete colored dots visualizing the winner-takes-all competition, indicating which thoughtseed has the highest activation at each moment. **Level 3—Meta-Awareness** (**top**): A purple line depicting meta-awareness fluctuations in response to changes in dominant thoughtseeds and meditation states. **Novice vs. Expert Differences: Activation Stability:** Experts exhibit more consistent thoughtseed activations, particularly for breath_focus and equanimity, with less noise than novices. **Dominance Patterns:** Novices display rapid switches in dominant thoughtseeds, reflecting frequent distractions, while experts sustain longer periods of breath_focus dominance with fewer interruptions. **Meta-Awareness Fluctuations:** Novices show more pronounced meta-awareness drops during mind-wandering, whereas experts maintain a higher baseline, even amidst distractions [39].

This hierarchical coupling (Figure 8) between thoughtseed activations, dominant thoughtseeds, and meta-awareness enables emergent state transitions, moving beyond predetermined sequences to capture the naturalistic dynamics of meditation [38,39].

### 4.3. Results Summary

This study has effectively demonstrated that the Thoughtseeds Framework accurately models the complex dynamics of Vipassana meditation across novice and expert meditators, elucidating the interplay of thoughtseed activations, dominant thoughtseed competition, and meta-awareness within a hierarchical system. The simulation replicates experience-dependent variations with precision, including enhanced attentional stability, reduced mind-wandering episodes, and improved meta-awareness in experts, which are consistent with empirical meditation research [36,38,79]. These findings corroborate the existing meditation literature, which emphasizes the development of sustained present-moment awareness and emotional regulation through practice [78,81]. Finally, the breath_control state, initially represented as explicit knowledge within a KD for novice meditators, transitions into an implicit, automatized process in experts through sustained practice, reflecting a shift from declarative to procedural processing [79].

## 5. Discussions

### 5.1. Thoughtseeds and Broadcasting Dynamics Within the Global Workspace

The Thoughtseeds Framework conceptualizes thoughtseeds as attentional agents that integrate information from knowledge domains (KDs) to shape cognitive states (Section 2). Within Global Workspace Theory (GWT) [44,45,46,47], thoughtseeds function as information processors competing for access to the ‘Global Workspace’, a central hub facilitating communication across specialized cognitive units. Through a winner-takes-all dynamic, the dominant thoughtseed at each timestep influences attention, decision-making, and behavior, ensuring a unitary conscious experience [45,46]. This process aligns with GWT’s principle of discrete conscious states, wherein conscious cognition manifests as a sequence of transient attractor states emerging from coordinated neural activity [47,84]. Continuous competition among thoughtseeds maintains a coherent stream of cognitive experience, preventing fragmented awareness [44]. The Intrinsic Ignition Framework (IIF) further elucidates these transitions as spontaneous ignition events—transient bursts of neural activity that drive state changes [84]. By balancing epistemic (information-seeking) and pragmatic (goal-directed) affordances through active inference, thoughtseeds guide adaptive behavior while minimizing surprise [62,64], thus supporting a coherent cognitive experience [46].

### 5.2. Towards a General Theory of Embodied Cognition

The Thoughtseeds Framework, grounded in neuronal packets [32,33,34] and the Free Energy Principle (FEP) [15], provides a foundation for advancing a general theory of embodied cognition [1,2,49]. Thoughtseeds propose higher-order constructs from the coordinated activity of knowledge domains (KDs), which encapsulate knowledge derived from neuronal packets and their superordinate ensembles (Section 2.1).

Within the context of our Vipassana meditation simulation, thoughtseeds function as attentional agents. In the current simulation, we only demonstrated thoughtseeds competing with each other, by establishing a one-to-one correspondence between thoughtseeds and knowledge domains. It is a simplification of the highly complex multi-scale organization of the brain [23,24,25]. In the simulation, thoughtseeds drive cyclical transitions between states, such as breath_control, mind_wandering, meta-awareness, and redirect_breath—reflecting how cognition arises from the interplay between the living system, its body, and the environment [8,9]. For instance, when breath_focus predominates, it sustains focused attention, reinforcing the breath_control state, whereas a shift to distraction thoughtseeds such as pain_discomfort or pending_tasks may lead to mind_wandering, as frequently observed in novice practitioners (Section 4.3). Meta-awareness modulates these transitions, facilitating recovery through states such as redirect_breath, a process that is more efficient in expert practitioners [38]. These self-organizing dynamics demonstrate the framework’s capacity to model thought processes as embodied and situated, offering a novel perspective on cognitive emergence.

### 5.3. Limitations and Future Research Directions

The Thoughtseeds Framework offers a novel approach to modeling thought dynamics in meditation (Section 3 and Section 4) as a starting point, but several limitations warrant further investigation. A key strength and intentional focus of this framework is modeling the phenomenology of thought dynamics—how thoughts arise, compete, and transition—as demonstrated in our simulation. However, this focus on modeling the movement of thoughts within a defined cognitive architecture means the framework does not currently aim to provide a comprehensive theory of consciousness itself. For instance, initial considerations of complex concepts like ‘Pure Awareness’ [41,42,43] were refined to maintain model tractability and focus on the core dynamics of attentional agents (thoughtseeds).

Mapping thoughtseed dynamics to specific neural correlates remains challenging because of the brain’s distributed networks. Although our framework identifies thoughtseeds as attentional agents (Section 2), linking their activity to precise brain regions (e.g., frontoparietal or default mode networks) requires advanced neuroimaging techniques [91]. Additionally, declarative KDs that involve conscious thought and memory elements, such as those supporting explicit knowledge and conscious recall, engage hippocampal and subcortical regions, further complicating the mapping process [58]. This increased complexity underscores the importance of understanding the phenomenology of these states, as phenomenological insights are crucial for accurately modeling the intricate dynamics of thoughtseed interactions across these regions [41]. Future research could leverage multimodal imaging (e.g., fMRI, EEG) to identify spatiotemporal patterns or oscillatory dynamics associated with thoughtseed transitions, enhancing the framework’s biological plausibility [95].

The framework currently focuses on a constrained set of meditation states (breath_control, mind_wandering, meta-awareness, and redirect_breath), limiting its generalizability to the brain’s vast repertoire of states. Extending the model to other cognitive contexts, such as creative problem-solving or emotional regulation, could broaden its applicability while addressing individual variability in cognitive styles [39]. Additionally, incorporating hierarchical interactions between thoughtseeds and KDs, potentially using nested Markov blanket structures, could better capture the multi-scale dynamics of cognition [34].

### 5.4. Key Limitations

#### 5.4.1. Metastability of Thoughtseeds

Thoughtseed dynamics exhibit rapid, metastable transitions between states (e.g., from breath_focus to pain_discomfort), complicating the identification of stable attractors representing core knowledge or behaviors (Section 4.2.4). This metastability poses challenges for empirical measurement and computational modeling, as tracking these transitions requires high temporal resolution and sophisticated analytical techniques [85,90].

#### 5.4.2. Hierarchical Complexity

The nested structure of thoughtseeds and knowledge domains (KDs) introduces complexity to understanding how higher- and lower-order processes interact (Section 2). For instance, meta-awareness modulates thoughtseed competition (Section 4.2), but capturing the interplay between these levels experimentally, particularly when linked to neurobiological signatures, remains difficult [36]. Future experimental designs could focus on specific meditation tasks to constrain this complexity and improve empirical precision.

#### 5.4.3. Individual Variability

Although the simulation captures differences between novices and experts (Section 4.3), broader individual variations in cognitive styles and meditation approaches remain unexplored [39]. Nevertheless, the Thoughtseeds Framework has the potential to address such variability in principle, as its self-organizing principles enable thoughtseeds to adapt to individual-specific KDs and activation patterns (Section 2). Future studies should investigate this by modeling thoughtseed dynamics tailored to diverse populations, thereby enhancing the generalizability of the framework.

### 5.5. Future Directions

Future research can further develop and validate the Thoughtseeds Framework by focusing on several key areas that build on our current meditation simulation (Section 3 and Section 4). Validating, refining, and fitting the model using specific real-world neuroimaging or behavioral datasets represents an important next step.

#### 5.5.1. Computational Modeling

The simulation employs discrete timesteps, which may oversimplify the continuous neural dynamics of meditative states (Section 5.3). Future work could develop continuous-time computational models to capture the gradual attentional shifts observed in mindfulness practices [36,76]. Simulating thoughtseed transitions as dynamic trajectories in a multi-dimensional state space, utilizing frameworks such as Leading Eigenvector Dynamics Analysis (LEiDA) [87], could enable the alignment of thoughtseeds (e.g., breath_focus, and meta-awareness) with specific recurrent brain states, such as those identified in Yeo subnetworks (e.g., doral attention, saliency networks), which have been extensively studied in meditation research [97]. This approach would facilitate testable hypotheses regarding thoughtseed dynamics, such as their role in sustaining focused attention or modulating mind-wandering in novices versus experts (Section 4.3), potentially elucidating neural correlates of meditation-induced cognitive changes [80].

Future theoretical work could refine the model’s phenomenological grounding by exploring concepts like a dynamic ‘sense of self’ [98] in non-meditative contexts, potentially offering alternatives to aspects described as ‘Pure Awareness’ in meditation literature [41,42,43]. Comparing the framework’s scope and mechanisms with broader theories, such as IWMT [99]—which integrates Integrated Information Theory [100] and Global Neuronal Workspace Theory [47] with the Free Energy Principle and Active Inference—or the Inner Screen Hypothesis [51], could also prove valuable.

#### 5.5.2. Computational Modeling of Diverse Meditation Paradigms

The Thoughtseeds Framework currently models focused attention (FA) meditation through states such as breath_control (Section 4.2). Future research could extend the framework to develop a comprehensive model encompassing focused attention, open monitoring (OM), and non-dual awareness (NDA)—three well-established meditation paradigms [40]. By adapting thoughtseed dynamics to reflect the distinct attentional mechanisms of each paradigm (e.g., sustained focus in FA, broad monitoring in OM, and non-conceptual awareness in NDA), the framework could potentially unify these practices under a single model, offering a comprehensive understanding of meditative cognition.

#### 5.5.3. Cognitive Development

The Thoughtseeds Framework has the potential to enhance our understanding of cognitive development by examining the emergence and evolution of thoughtseeds during learning and skill acquisition. Future research could apply this framework to focused attention tasks in developmental contexts, such as numerical cognition [101], to investigate the development of thoughtseeds representing numerical concepts over time. This approach could provide valuable insights into the influence of meditative practices on cognitive processes across a lifespan, building upon our findings of enhanced attentional stability in expert meditators (Section 4.3).

#### 5.5.4. Clinical Applications

Investigating the Thoughtseeds Framework’s potential in clinical contexts could elucidate how disruptions in thoughtseed dynamics contribute to attention disorders such as ADHD. For instance, the frequent mind-wandering observed in novices (Section 4.3) may parallel attention lapses in clinical populations, suggesting that interventions targeting thoughtseed regulation could enhance cognitive function. Further research could examine whether mindfulness-based interventions, which improve meta-awareness in our simulation (Section 4.2), can be adapted to regulate thoughtseed dynamics in clinical settings [39].

The Thoughtseeds Framework presents a biologically grounded model of cognition, elucidating the emergence of thoughtseeds from the interaction between neuronal packets, KDs, and higher-order processes (Section 2). Through these research directions, the framework can be refined and validated, potentially offering more profound insights into cognition, conscious experience, and their underlying neural mechanisms within meditative and clinical contexts.

## 6. Conclusions

This study establishes the Thoughtseeds Framework as an effective, biologically grounded model for simulating thought dynamics in Vipassana meditation, laying a foundation for advancing neuroscience research into the content of consciousness. Our simulation highlights distinct novice–expert differences: experts maintain control dominance of sustained focused attention, whereas novices exhibit more frequent mind-wandering episodes, aligning with empirical findings on attentional stability [38].

By simulating the interplay between knowledge domains, competing thoughtseeds, and meta-cognition, this work offers a computationally grounded approach to understanding the *phenomenology of thought dynamics* and state transitions in meditation. While distinct in scope from comprehensive consciousness theories, the Thoughtseeds Framework provides a specific, mechanistic tool for investigating how subjective experiences of attention and distraction emerge from underlying dynamics, offering a foundation for future research across cognitive science and clinical domains. Viewed through Global Workspace Theory, thoughtseeds coordinate a cohesive meditative experience, balancing epistemic and pragmatic affordances through active inference. The framework’s emphasis on embodied cognition further illustrates how thought processes originate from the dynamic interplay among the practitioner, their body, and the environment.

By integrating computational modeling with phenomenological insights, the Thoughtseeds Framework serves as a robust research tool to elucidate the nature of mind and thought in a comprehensive manner, providing a biologically plausible perspective that extends beyond meditation to encompass diverse cognitive processes and clinical conditions.

## Figures and Tables

**Table 1:** Neuronal Packet interactions.

Concept	Explanation
Neuronal Packet (NP)	Based on the Free Energy Principle, a self-organizing ensemble of neurons that encodes a specific feature or aspect of the world.
Encapsulated Knowledge Structure	The structured knowledge content within an NP’s Markov Blanket associated with its core attractor.
Superordinate Ensemble (SE)	A higher-order organization emerging from the coordinated activity of multiple NPs, via a shared generative model, enabling the representation of more complex and abstract concepts.
Core Attractor	The most probable and stable pattern of neural activity within a manifested NP, or a higher-order SE, embodying its core functionality or the core knowledge structure.

**Table 2:** Key concepts of the Thoughtseeds Framework.

Concept	Explanation
Knowledge Domain (KD)	Self-organizing units of embodied knowledge, akin to metastable brain states, encapsulating neuronal packets (NPs) or ensembles, forming the neural basis for conscious and unconscious processing.
Thoughtseed	Dynamic attentional agents intrinsic to a specific KD, which acts as its core attractor. It represents recurring neural patterns associated with specific concepts, percepts, or actions, competing for the *attention spotlight*.
Global Activation Threshold	A dynamic threshold shaped by attention and arousal, setting the minimum activation for thoughtseeds to enter the active pool and compete for dominance.
Active Thoughtseed Pool	Thoughtseeds exceeding the global activation threshold, forming a pool of candidate attentional agents competing to influence conscious content in the Global Workspace.
Dominant Thoughtseed	The thoughtseed with the highest activation, minimizing Expected Free Energy, which enters the Global Workspace via winner-takes-all dynamics to shape consciousness and guide attention. It currently holds the attention spotlight.
Meta-awareness Parameter	A meta-cognitive parameter reflecting the brain’s self-monitoring, modulating thoughtseed competition and attentional precision in the Global Workspace.
Attention Precision	A meta-cognitive parameter enhancing selective attention, prioritizing thoughtseeds to gain the attention spotlight, influence their dominance in the Global Workspace, and shape conscious content.

## Data Availability

The code is available in GitHub repository https://github.com/prakash-kavi/thoughtseeds_vipassana.

## Supplementary Materials

The following supporting information can be downloaded at https://www.mdpi.com/article/10.3390/e27050459/s1, S1. Descriptions of the Mathematical Equations.
