# Supplementary material for: Thoughtseeds: A Hierarchical and Agentic Framework for Investigating Thought Dynamics in Meditative States
Source: Entropy (Basel). 2025 Apr 24;27(5):459. doi: 10.3390/e27050459 (PMC12110063; doi:10.3390/e27050459)
Supplement: Supplementary file 1 [file entropy-27-00459-s001.zip › entropy-3542628-supplementary S1.pdf]

Supplementary Section

1. Descriptions of the Mathematical Equations

1.1 Learning Equations

$$W_{ts,\text{state}} = \begin{cases} w_{base} \cdot U(0.9, 1.1) & \text{if } ts \in \text{primary attractor for state} \\ w_{base} \cdot U(0.7, 0.9) & \text{if } ts \in \text{secondary attractor for state} \\ w_{base} \cdot U(0.05, 0.2) & \text{otherwise} \end{cases} \tag{1}$$

where:

- w_{base} represents experience-dependent baseline weights (e.g., breath_focus: 0.8 novice, 0.95 expert)
- $U(a, b)$ adds biologically plausible variability through uniform random sampling
- Primary attractors (e.g., breath_focus for breath_control) receive strongest weights
- Weights are clipped to biologically plausible range [0.05, 1.0]

$$P(\text{State}_t \rightarrow \text{State}_{t+1}) = \begin{cases} 1 & \text{if } \phi(\text{State}_t, \boldsymbol{\alpha}_t) > \theta_{\text{transition}} \text{ and } \text{dwell}_t \geq \text{dwell}_{\text{min}} \\ 0 & \text{otherwise} \end{cases} \tag{2}$$

where:

- $\phi(State_t, \boldsymbol{\alpha}_t)$ evaluates specific activation patterns (distraction_level = pain_distraction + pending_tasks for focused states; self_reflection for mind wandering)
- $\theta_{transition}$ represents critical threshold values (mind_wandering: 0.25, meta_awareness: 0.30, redirect_breath: 0.30)
- t_{dwell} ensures minimum state duration for biological plausibility

$$\boldsymbol{\alpha}_{t+1} = (1-\gamma) \cdot \boldsymbol{\alpha}_t + \gamma \cdot \boldsymbol{\alpha}_t^{target} + \Delta\boldsymbol{\alpha}_t^{dist} + \boldsymbol{\eta}_t \tag{3}$$

where:

- Neural momentum parameter $\gamma = 0.9$ (10% inertia from previous state)
- $\boldsymbol{\alpha}_t^{target}$ is the target activation pattern for the current state and is determined by learned weights, meta-awareness, and interactions
- $\boldsymbol{\eta}_t \sim \mathcal{N}(0, \sigma_{\text{exp}})$ adds biological variability (novice: 0.08, expert: 0.04)

The distraction growth component $\Delta\boldsymbol{\alpha}_t^{dist}$ introduces nonlinear dynamics:

$$\Delta\alpha_{i,t}^{\text{dist}} = \begin{cases} \beta \cdot d_t \cdot \lambda_{\text{exp}} \cdot \xi_t & \text{if thoughtseed } i \in \{\text{pain\_discomfort, pending\_tasks}\} \\ -\phi \cdot d_t \cdot \frac{t_{\text{focused}}}{10} & \text{if thoughtseed } i = \text{breath\_focus} \\ 0 & \text{otherwise} \end{cases}$$

where:

- base distraction growth rate $\beta = 0.035$
- $d_t = \min(1.0, \frac{\text{dwell}_t}{\max(10, \text{dwell}_{\text{limit}})})$ is the nonlinear dwell factor. The dwell-dependent growth factor creates a sigmoidal growth curve for distractions consistent with leaky accumulator models of attention
- Experience-dependent scaling λ_{exp}(2.5 for novices, 1.2 for experts). Differential distraction susceptibility between novices and experts (λ_exp = 2.5/1.2) models neuroplasticity effects documented in long-term meditators [36].
- Random distraction spike factor ξ_t (10% chance of 3 times strength) Heavy-Tailed Distraction Distribution: The random spike mechanism with 10% probability of 3× distraction strength implements the "heavy-tailed" distribution of thought intrusions (pending_tasks and pain_discomfort) observed in meditation
- Attentional Fatigue rate ϕ (0.01 for novices, 0.005 for experts) The gradual fatigue in focus elements implements resource depletion models from cognitive psychology
- $t_{focused}$ tracks cumulative time in focused states

State-Specific Activation Modulation:

During mind wandering with low meta-awareness:

- breath_focus activation multiplied by 0.05 (strong suppression)
- distraction thoughtseeds multiplied by 1.2 (enhancement)
- other thoughtseeds multiplied by 0.5 (moderate suppression)

Interaction Networks with Feedback

$$\alpha_{ts,t}^{\text{target}} = W_{ts,\text{state}} \cdot (1 + \mu_t \cdot 0.1) + \sum_{j \neq ts} I_{ts,j} \cdot \alpha_{j,t} \cdot \frac{\mu_{\text{state}}}{30}$$

where

- The Interaction matrix $I_{ts,j}$ creates inhibitory/excitatory feedback loops similar to those in recurrent neural networks, , modeling lateral inhibition and facilitatory connections in attentional networks. Interaction strengths between thoughtseeds ranging from -0.6 to +0.7)
- $\frac{\mu_{state}}{30}$ is the dwell time scaling factor (normalizing by max mean dwell)

$$\mu_t = \begin{cases} \max(0.55, 0.6 - 0.05 + \epsilon_t) & \text{if state} = \text{mind\_wandering and dominant\_ts} \in \{\text{distractions}\} \\ \max(0.6, 0.6 + \epsilon_t) & \text{if state} = \text{mind\_wandering, other cases} \\ \min(\mu_{\max}, 0.6 + 0.4 + \epsilon_t) & \text{if state} = \text{meta\_awareness} \\ \min(0.85, 0.6 + 0.25 + \epsilon_t) & \text{if state} = \text{redirect\_breath} \\ \min(\mu_{\max}, 0.6 + 0.2 + \epsilon_t) & \text{if state} = \text{breath\_control} \end{cases} \quad (4)$$

where:

- Base awareness level = 0.6

- State-specific adjustments range from -0.05 to +0.4
- $\epsilon_t \sim \mathcal{N}(0, \sigma_{\text{exp}}/2)$ adds reduced noise for stability by experience level
- μ_{max} is 1.0 for experts and 0.9 for novices in meta-awareness state, and it varies based on state

$$T_{i,j} = \frac{\text{count}(\text{State}_i \to \text{State}_j)}{\text{total\_transitions\_from\_State}_i} \tag{5}$$

And captures mean activation patterns at transition points:

$$\bar{\boldsymbol{\alpha}}_{i,j}^{\text{trans}} = \frac{1}{n_{i,j}} \sum_{k=1}^{n_{i,j}} \boldsymbol{\alpha}_{i \to j}^{(k)} \tag{6}$$

where:

- $n_{i,j}$ is the number of transitions from state *i* to state *j*
- $\boldsymbol{\alpha}_{i \to j}^{(k)}$ is the activation pattern at the *k*-th transition from state *i* to *j*

1.2 Simulation Equations

$$\mathcal{G}_{x \to y} = \sum_{l=1}^{L} \frac{RSS(y|y_{past}) - RSS(y|y_{past}, x_{past})}{RSS(y|y_{past}, x_{past})} \tag{7}$$

where RSS represents residual sum of squares from the regression models, and *L* represents the maximum lag (5 timesteps).

$$\mathcal{S}_{x \to y} = (1 - p_{min}) \cdot \text{sign}(r_{xy})$$

where p_{min} is the minimum p-value across all lags and r_{xy} is the Pearson correlation determining the sign of interaction.

$$\alpha_i(t+1) = r_i \cdot \text{Target}_i(t) + (1 - r_i) \cdot \alpha_i(t) \tag{8}$$

The responsiveness parameter r_i represents expertise-dependent neuroplasticity.

$$\text{Target}_i(t) = W_i^s + \sum_{j \neq i} W_{ij} \cdot \alpha_j(t) \cdot \tau + \gamma_i^s \cdot \tau + m(t) \cdot \beta_i \tag{9}$$

where:

- W_i^s is the state specific base activation. It refers to the intrinsic activation level of a thoughtseed independent of influences from other thoughtseeds
- W_{ij} is the inter-thoughtseed interaction weights
- $\alpha_j(t)$ is the activation level of the other thoughtseed at time *t*
- γ_i^s is the state specific growth-term
- $m(t)$ is the meta-awareness term
- β_i is the meta-awareness sensitivity

$$P(\text{State}_{t+1} = j | \text{State}_t = i, \boldsymbol{\alpha}_k(t)) = f(T_{ij}, \boldsymbol{\alpha}_k t, \theta_k) \tag{10}$$

where:

- T_{ij} is the base transition probability from state *i* to *j*
- $\boldsymbol{\alpha}_k(t)$ is the set of all thoughtseed activations at time *t*
- θ_k is the set of transition thresholds
- f is the transition function incorporating both probabilistic and deterministic elements

Dominant Thoughtseed Dynamics in the Hierarchical Framework

This selection process reflects the winner-take-all dynamic within the Global Workspace, where the most activated thoughtseed gains prominence and influences conscious awareness [46]. This dominant thoughtseed then influences:

$$P(\text{State}_{t+1} = j | \text{State}_t = i, \boldsymbol{\alpha}_k(t)) = f(T_{ij}, \boldsymbol{\alpha}_k t, \theta_k) \quad (11)$$

State transition probabilities: When a dominant distraction thoughtseed's activation exceeds its threshold (θ_mind_wandering), it increases the probability of transitioning to a mind-wandering state.

$$P(\text{State}_{t+1} = \text{mind\_wandering}) \propto \max(\alpha_{\text{pain\_discomfort}}(t), \alpha_{\text{pending\_tasks}}(t)) > \theta_{\text{mind\_wandering}}$$

Meta-awareness modulation: Meta-awareness m(t) responds differently to different dominant thoughtseeds - remaining low during distraction dominance but increasing rapidly when self-reflection becomes dominant:

$$m(t{+}1) = \begin{cases} m(t) \cdot (1 - \delta_{\text{decay}}) & \text{if Dominant}_t \in \{\text{pain\_discomfort, pending\_tasks}\} \\ \min(1.0, m(t) + \delta_{\text{boost}}) & \text{if Dominant}_t = \text{self\_reflection} \end{cases}$$

Network synchronization: The dominant thoughtseed synchronizes network activity around its activation pattern, creating transient attractor states similar to those observed in neural synchronization studies [80].

Let $\mathcal{T} = \tau_1, \tau_2, \ldots, \tau_n$ represent the set of thoughtseeds (e.g., breath_focus, pain_discomfort, self_reflection), with each thoughtseed τ_i having an activation level $\alpha(\tau_i, t)$ at timestep *t*. These activation levels are determined by the thoughtseed network dynamics (Section 4.2.2, Equation 8). The dominant thoughtseed at timestep *t*, denoted $\boldsymbol{\tau}^*(t)$, is the thoughtseed with the highest activation:

$$\boldsymbol{\tau}^*(t) = \arg\max_{\tau_i \in \mathcal{T}} \boldsymbol{\alpha}(\tau_i, t) \quad (12)$$
